# Challenges in the reproducibility of clinical studies with resting state fMRI: An example in early Parkinson's disease

**DOI:** 10.1016/j.neuroimage.2015.09.021

**Published:** 2016-01-01

**Authors:** Ludovica Griffanti, Michal Rolinski, Konrad Szewczyk-Krolikowski, Ricarda A. Menke, Nicola Filippini, Giovanna Zamboni, Mark Jenkinson, Michele T.M. Hu, Clare E. Mackay

**Affiliations:** a Centre for the functional MRI of the Brain (FMRIB), University of Oxford, Oxford, UK; b Oxford Parkinson's Disease Centre (OPDC), University of Oxford, Oxford, UK; c Nuffield Department of Clinical Neurosciences, University of Oxford, Oxford, UK; d Department of Psychiatry, University of Oxford, Oxford, UK

**Keywords:** Resting state functional magnetic resonance imaging (rfMRI), Functional connectivity, Artefact removal, Dual regression, Basal ganglia network, Parkinson's disease

## Abstract

Resting state fMRI (rfMRI) is gaining in popularity, being easy to acquire and with promising clinical applications. However, rfMRI studies, especially those involving clinical groups, still lack reproducibility, largely due to the different analysis settings. This is particularly important for the development of imaging biomarkers. The aim of this work was to evaluate the reproducibility of our recent study regarding the functional connectivity of the basal ganglia network in early Parkinson's disease (PD) (Szewczyk-Krolikowski et al., 2014). In particular, we systematically analysed the influence of two rfMRI analysis steps on the results: the individual cleaning (artefact removal) of fMRI data and the choice of the set of independent components (template) used for dual regression.

Our experience suggests that the use of a cleaning approach based on single-subject independent component analysis, which removes non neural-related sources of inter-individual variability, can help to increase the reproducibility of clinical findings. A template generated using an independent set of healthy controls is recommended for studies where the aim is to detect differences from a “healthy” brain, rather than an “average” template, derived from an equal number of patients and controls. While, exploratory analyses (e.g. testing multiple resting state networks) should be used to formulate new hypotheses, careful validation is necessary before promising findings can be translated into useful biomarkers.

## Highlights

• Reproducibility of clinical findings is crucial for imaging biomarker development.
• We addressed the impact on reproducibility of different analysis settings in rfMRI.
• ICA-based cleaning of rfMRI data increases reproducibility.
• The effect of the template choice for dual regression is evaluated.

## Introduction

Resting state functional MRI (rfMRI) has been shown to be a promising tool for exploring brain functions and assessing their alteration in neurodegenerative conditions (Barkhof et al., 2014). Over the last decade, several resting state networks (RSNs) have been identified (Beckmann and Smith, 2004, Smith et al., 2009) and associated with specific brain functions through the comparison with results obtained from task-based fMRI experiments (Smith et al., 2009, Zamboni et al., 2013). Moreover, rfMRI has been shown to be stable across subjects (Smith et al., 2009, Zuo and Xing, 2014), easy to acquire, and as it is not dependent on task performance, functional connectivity (FC) of the RSNs can be evaluated in impaired subjects. Therefore, rfMRI has become a common technique in clinical research studies. With observed alterations of RSNs now reported in subjects with clinical symptoms and increased at-risk of developing pathology (Barkhof et al., 2014, Filippini et al., 2009, Sole-Padulles et al., 2013), rfMRI may have a vital role in the development of novel imaging biomarkers.

Despite the importance of obtaining reliable and stable results that may be later used as biomarkers, rfMRI studies, especially those involving clinical groups, still lack reproducibility. In fact, even when reproducibility tests are performed, they are usually performed on healthy controls, and issues may only become apparent when dealing with patient groups. For example, logistical difficulties may arise from subjecting patients to long or multiple scanning sessions. Moreover, in clinical studies, images are typically acquired using clinical scanners. This may result in poorer data quality, leading to suboptimal processing steps, such as registration and artefact removal. Importantly, the most reproducible networks (default, control and attention networks—see Zuo and Xing, 2014) may not necessarily be the ones that are of the greatest clinical importance. For example, although only recently described (Robinson et al., 2009) and, therefore, not studied in great detail, the basal ganglia network (BGN) has recently been shown to be affected in early PD (Szewczyk-Krolikowski et al., 2014).

In addition to the paucity of within-group test–retest reliability (Zuo and Xing, 2014), the lack of reproducibility between studies may be due to the different analysis settings, with a major contributor being the many permutations in analysis pipelines. In a fast moving field of rfMRI, there is continual development and refinement of methodology. Several studies evaluated the impact of analysis methods on the reproducibility and reliability of RSNs (Franco et al., 2013, Zuo et al., 2010, Zuo and Xing, 2014). Specifically, it has been demonstrated that independent component analysis (ICA), and in particular group-ICA followed by dual regression, rather than single-subject ICA and template matching (Zuo et al., 2010), is more stable than seed-based analysis (Zuo and Xing, 2014). However, even within these guidelines, there are several analytical details that can influence the results and make comparisons difficult.

In light of these observations, we endeavoured to evaluate the reproducibility of our recent study of functional connectivity within the BGN of patients with early PD (Szewczyk-Krolikowski et al., 2014). The difference observed in the BGN connectivity was substantial in both magnitude and extent and therefore provides a good test-bed. In particular, we systematically analysed the influence of two rfMRI analysis steps: the individual cleaning (artefact removal) of fMRI data and the choice of a RSNs template (a set of independent components) within the framework of dual-regression ICA. The aim of this work was to establish how strongly the settings of these steps affected the observed results. We hoped to aid interpretations and comparisons across studies and contribute to the translational pipeline for reliable imaging clinical biomarkers.

## Materials and methods

### Participants

Fifty-nine patients with PD (mean age = 63.2 ± 10.9 years, F:M = 25:34) and thirty age- and gender-matched healthy controls (HC) (mean age = 62.8 ± 7.2, F:M = 14:16) were recruited from the Oxford Parkinson's Disease Centre (OPDC) cohort (Rolinski et al., 2014). This sample includes the cohort described in Szewczyk-Krolikowski et al. (2014). Patients included in the PD group met the UK PD Society Brain Bank Criteria for clinically probable idiopathic PD (Hughes et al., 1992), having predominantly akinetic-rigid parkinsonism with minimal tremor. Patients taking dopaminergic medications were scanned in a clinically defined “off-state,” a minimum of 12 hours after the withdrawal of their relevant medications. Subjects included in the HC group had no family history of parkinsonism and were recruited largely from the spouses and friends of the PD participants. All participants underwent a detailed clinical assessment (Szewczyk-Krolikowski et al., 2014). Both groups only included subjects classified as cognitively healthy, as defined by a Mini-Mental State Examination (MMSE) > 26 (Folstein et al., 1975) and no subjective complaint of memory problems.

Each subject gave written consent to participate in the study, which was conducted with the approval of the local NHS ethics committee and in compliance with national legislation and the Declaration of Helsinki.

### Neuroimaging data acquisition and preprocessing

Scanning was performed at the Oxford Centre for Clinical Magnetic Resonance Research (OCMR) using a 3 T Trio Siemens MRI scanner (Erlangen, Germany) equipped with a 12-channel head coil. The protocol included 1) high-resolution T1-weighted images (MPRAGE, resolution 1 × 1 × 1 mm^3^, TE/TR = 4.7 ms/2040 ms, 192 axial slices, 6 minutes); 2) rfMRI images (EPI, resolution 3 × 3 × 3.5 mm^3^, TE/TR = 28 ms/2000 ms, 34 axial slices per volume, covering both hemispheres with incomplete coverage of the cerebellum, 180 volumes in 6 minutes, eyes open); 3) field map images, to account for distortions caused by field inhomogeneities (GRE, resolution 3 × 3 × 3.5 mm^3^, TR = 488 ms, TE = 5.19 ms and 7.65 ms).

The analysis of resting state fMRI data was performed using FSL software package (Jenkinson et al., 2012). Firstly, images were motion corrected with MCFLIRT; from this operation, the six rigid-body parameter time series were extracted for each subject (to be used for subsequent cleaning) and the mean relative displacement was calculated to ensure that the two groups were matched in terms of average amount of head motion (HC: 0.14 ± 0.09 mm; PD: 0.12 ± 0.05 mm, p = 0.23). Following preprocessing steps included brain extraction, unwarping using fieldmap data, spatial smoothing using a Gaussian kernel of FWHM of 6 mm, and high-pass temporal filtering of 150 s. Single-subject probabilistic independent component analysis (ICA) was then performed with MELODIC tool (Beckmann and Smith, 2004) with automated dimensionality estimation to be used for ICA-based artefact removal.

T1-weighted images were brain-extracted and used as anatomical references for fMRI. Tissue segmentation was also performed with FAST (Zhang et al., 2001) and the grey matter (GM) images were registered to the MNI 152 standard space using non-linear registration with FNIRT and used to generate voxel-wise confound regressors for fMRI statistical analyses.

### Reproducibility analyses of resting state fMRI data

#### Analyses overview

In this work, we aimed to systematically analyse the influence of two rfMRI analysis steps: (1) the individual cleaning (artefact removal) of fMRI data and (2) the choice of the set of independent components used as input for dual regression (from now on referred as *template*).

The impact of artefact removal was tested on a subsample of 19 HC and 19 PD (matched for age, sex, and head motion) of our cohort, specifically the same subjects used in Szewczyk-Krolikowski et al. (2014), comparing six cleaning options (see Section 2.3.2. for details). The rationale for using this subsample for this first analysis is that we judged it to be sufficiently large to test differences among the different approaches, while limiting the manual intervention (in terms of both expertise and time) required for hand-labelling the single-subject components (used as gold standard cleaning method). Firstly, we tested the effect of cleaning on the temporal signal-to-noise ratio, which should be higher with better cleaning. Subsequently, we calculated spatial correlations between the subject-specific BGN maps (derived with dual regression) obtained after each cleaning approach with respect to a gold standard (the BGN maps obtained with manual cleaning, see Section *Influence of artefact removal*). A higher spatial correlation corresponds to a better cleaning approach. In order to compare the effect of cleaning on between-group discriminability, we performed a regions-of-interest (ROI) analysis and a voxel-wise analysis of the BGN. We then repeated the comparison, among the automated methods only, on the full sample (30 HC and 59 PD, which included the subsample described above) to verify that the results obtained in the subsample were consistent and reproducible with respect to sample size.

Secondly, the impact of the template used for dual regression was tested on the whole cohort of 30 HC and 59 PD, comparing six templates (see Section *Influence of template for dual regression* for details). Similarly to the analyses carried out to compare the effect of the cleaning approaches, we evaluated the impact of the template choice on between-group discriminability by performing an ROI analysis and a voxel-wise analysis of the BGN, also quantifying the level of similarity/overlap among the results of the voxel-wise analyses.

Additionally, to ensure that our results were not influenced by the sample composition, we randomly split the full sample 100 times into two group pairs of PD patients and HC, repeated the analyses with different cleaning methods and the templates, and calculated the reproducibility across groups' composition. The detailed methods and results relative to this analysis are described in the supplementary material.

#### Influence of artefact removal

To remove the effect of motion, non-neural physiology, scanner artefacts, and other confounds, we applied a number of different cleaning options, each requiring different levels of manual intervention and expertise for the classification of signal and artefactual components. Subsequently, we tested the reproducibility of our previous findings with respect to these preprocessing step in the subsample of subjects described in Szewczyk-Krolikowski et al. (2014), including 19 HC and 19 PD.

In total, six datasets were obtained with the following cleaning approaches:

1) Uncleaned data: basic preprocessing only, as described in *Neuroimaging data acquisition and preprocessing* Section. No manual intervention or expertise required.
2) Motion regression: regression of 24 motion parameters (Satterthwaite et al., 2013) (six rigid-body parameter time series, their backward-looking temporal derivatives, and the squares of the twelve resulting regressors). No manual intervention or expertise required.
3) FIX standard 20: regression of 24 motion parameters, plus cleaning using the FIX tool (Salimi-Khorshidi et al., 2014). FIX automatically classified the components obtained after single-subject ICA (average number of components estimated per subject = 43.89 ± 7.19) into signal or noise using a standard training dataset provided with the tool (and default threshold) and removed the contribution of the artefactual components (average number of components removed per subject = 21.66 ± 7.12, corresponding to 48.67 ± 11.22 % of the total variance) regressing out from the data only the unique variance related to the artefacts (Griffanti et al., 2014). No manual intervention or expertise required.
4) FIX OPDC 5: regression of 24 motion parameters, plus cleaning using the FIX tool (Salimi-Khorshidi et al., 2014), trained on a study-specific sample of 50 subjects belonging to the OPDC cohort including both HC and PD patients and a threshold of 5 to balance between noise removal and signal loss, which gave an accuracy of 98.2% true-positive ratio (TPR) and 65.8% true-negative ratio (TNR) at leave-one-out test. With this training dataset, 21.79 ± 7.66 components per subject were removed (48.72 ± 10.33% of the total variance). Manual intervention and expertise required only for the generation of the training dataset.
5) FIX OPDC 10: regression of 24 motion parameters, plus cleaning using the FIX tool (Salimi-Khorshidi et al., 2014), trained on the study-specific sample of 50 subjects belonging to the OPDC cohort and a threshold of 10 (accuracy of 96.9% TPR and 72% TNR at leave-one-out test). With this training dataset, 23.84 ± 7.98 components per subject were removed (52.39 ± 9.97% of the total variance). Manual intervention and expertise required only for the generation of the training dataset.
6) Manual cleaning: regression of 24 motion parameters, plus removal of the contribution of the artefactual components manually identified after single-subject ICA (29.76 ± 7.54 components removed per subject, corresponding to 63.98 ± 9.29% of the total variance). Manual intervention and expertise required for each subject (average time for manual classification of independent components of one subject for a trained operator = 20 minutes).

For each cleaning approach, we first calculated a global measure of temporal signal-to-noise ratio (tSNR). A raw tSNR image was formed by dividing the mean image across time by the standard deviation image over time. The tSNR image was then eroded to exclude brain-edge effects, and the median tSNR value was calculated. A paired t-test was used to compare the median tSNR achieved using different cleaning approaches.

Cleaned data were then linearly registered to the corresponding structural image using FLIRT (Jenkinson et al., 2002), optimised using Boundary-Based Registration (Greve and Fischl, 2009), and registered to the MNI space using non-linear registration. The dual regression approach (Filippini et al., 2009) was used to identify individual temporal dynamics and the associated spatial maps of the BGN. In order to allow direct comparison across the different cleaning options, we used the same template used in (Szewczyk-Krolikowski et al., 2014). To generate this template, group-ICA (with dimensionality d = 50) was performed temporally concatenating data from 80 healthy elderly subjects (including 19 from the OPDC cohort and 61 healthy control scans from previously published studies (Filippini et al., 2011, Filippini et al., 2012, Zamboni et al., 2013). All data were acquired on the same scanner using an identical acquisition protocol. A subset of components including the basal ganglia network (BGN) and 21 components identified as artefactual was then used as template for dual regression.

Subsequently, we calculated spatial correlation between the subject-specific BGN maps (output of stage 2 of dual regression) obtained after each cleaning approach with respect to the ones obtained with manual cleaning (defined as the gold standard approach).

Then, in order to compare the effect of cleaning on between-group discriminability, we performed a regions-of-interest (ROI) analysis and a voxel-wise whole-brain analysis of the BGN. Mean parameter estimates (P.E.) were extracted from the subject-specific BGN spatial maps within ROIs corresponding to the caudate, pallidum, and putamen, bilaterally, and the obtained values were then compared between the two groups with a two-sample independent t-test, with Bonferroni correction for multiple comparison across structures.

Finally, voxel-wise differences in the BGN maps were tested using a non-parametric permutation test, covarying for age and voxel-wise GM. Results were considered significant for p < 0.05 after correction for multiple comparisons with TFCE approach.

Additionally, to test the reproducibility of our findings with respect to sample size, we repeated the ROI and voxel-based analyses in the full sample, comparing the automated methods only.

#### Influence of template for dual regression

Data from the whole sample of 30 HC and 59 PD were used to assess the variability of FC analyses performed with dual regression when changing the template used to derive subject-specific time series and spatial maps. In particular, we tested the influence of using a different number and/or type of subjects to create the template, comparing the main approaches used in literature (Schultz et al., 2014): the use of out-of-sample, a priori templates of healthy controls (Szewczyk-Krolikowski et al., 2014), or an equal number of patients and controls from the specific study (Filippini et al., 2011, Filippini et al., 2012, Zamboni et al., 2013). We also tested whether to include only the RSN of interest (plus the artefactual components) or to perform a more exploratory analysis including the full set of components as spatial regressors.

To this aim, group-ICA with temporal concatenation and a fixed number of 50 components (dimensionality empirically determined to be able to clearly identify the BGN as a separate component) was performed on the following datasets:

1) 80 HC: same template used in Szewczyk-Krolikowski et al. (2014), using 80 elderly healthy controls (19 from the OPDC cohort and 61 healthy control scans from previously published studies (Filippini et al., 2011, Filippini et al., 2012, Zamboni et al., 2013) that used the same scanner and acquisition protocol).
2) 45 HC: generated with data from 45 elderly healthy controls only from previously published studies (Filippini et al., 2011, Filippini et al., 2012, Zamboni et al., 2013), age- and sex-matched to both HC and PD. The two Filippini studies (Filippini et al., 2011, Filippini et al., 2012) selected participants on the basis of APOE genotype, and the information about APOE genotype was available for the participants in the study performed by Zamboni and colleagues (Zamboni et al., 2013), so we subsampled these individuals such that the proportion of ε4-allele carriers was in line with the prevalence in the average population (Menzel et al., 1983).
3) 30HC30PD (study-specific template): generated from 30 HCs and 30 randomly selected age- and sex-matched PDs from our study.

The whole output of each group-ICA constituted a template for dual regression. Moreover, three additional templates were created by only including the BGN and the artefactual components identified in each group-ICA output. In this way, we obtained and compared the following six templates: a) 80HC-BGN (i.e. BGN plus artefactual components), b) 80HC-ALL (i.e. keeping all 50 components), c) 45HC-BGN, d) 45HC-ALL, e) 30HC30PD-BGN, and f) 30HC30PD-ALL.

Once the best cleaning approach was identified in the previous analysis, all data were cleaned, coregistered to MNI space, and subject-specific BGN spatial maps were obtained for each of the templates described above.

Similarly to the analyses carried out to compare the effect of the cleaning approaches, we compared the mean P.E. extracted from the subcortical ROIs and performed a voxel-wise non-parametric permutation test, covarying for age and voxel-wise GM and masking for an average GM mask across subjects.

We also quantified the level of similarity among the results of voxel-wise analysis by calculating the spatial correlation among the statistical maps (t-maps) obtained with each template and, after generating binary images of the significant clusters obtained with each template (t-maps thresholded at p = 0.05 corrected for multiple comparisons with TFCE approach), we calculated the Dice index between each pair of images as 2*(number of overlapping voxels)/(sum of voxels in each image).

Finally, we performed an exploratory analysis on 80HC-ALL, 45HC-ALL, and 30HC30PD-ALL templates to investigate if and how the template used for dual regression would affect also the results from other RSNs (see supplementary material for details).

## Results

### Influence of artefact removal

**Fig. 1:**
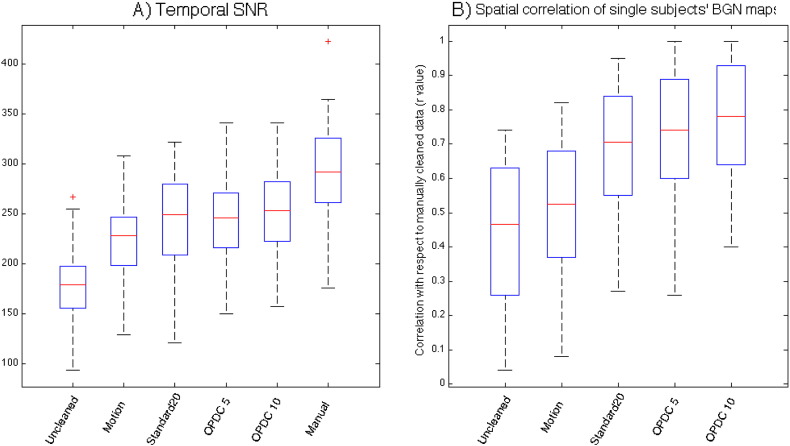
A) Temporal SNR of data obtained with different cleaning options. B) Spatial correlation between single-subject BGN maps, using the same template for dual regression as in Szewczyk-Krolikowski et al. (2014), obtained using data cleaned with different options and the corresponding map obtained using manually cleaned data (i.e. after removing the contribution of manually selected artefactual components after single-subject ICA).

The temporal SNR was significantly higher (p < 0.01) after cleaning, with smaller differences among the three different FIX options (Uncleaned < Motion regression < Standard 20 = OPDC 5 < OPDC 10 < Manual; see Fig. 1, panel A). SNR was not statistically different between the HC and PD groups with any of the approaches.

Spatial correlation analysis of subject-specific BGN maps with respect to the ones obtained with manual cleaning showed a significant increase with cleaning, especially when using ICA-based approaches (Uncl < Motion regression < Standard 20 = OPDC 5 < OPDC 10, Standard 20 < OPDC 10) (Fig. 1, panel B).

**Fig. 2:**
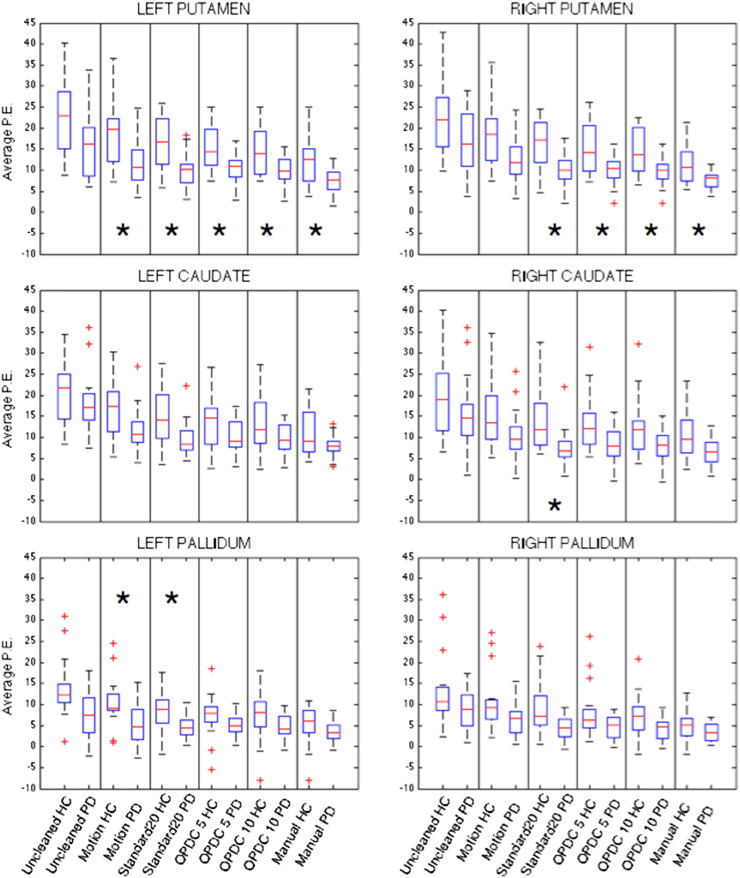
Average P.E. in the basal ganglia ROIs extracted from single-subject BGN maps obtained from data cleaned with different options. *Significant between-group differences surviving Bonferroni correction across structures.

The results of the ROI analysis in the basal ganglia are shown in Fig. 2 and Table 1. The average P.E. within the putamen was significantly lower in the PD group bilaterally after minimal cleaning. We obtained similar results with manual cleaning and the study-specific training dataset (i.e. significantly lower FC in PD compared to controls only in the bilateral putamen), while using the standard training dataset, we also observed between-group differences in FC (HC > PD) also within the right caudate and the left pallidum. The comparison across (automated) cleanings on the full sample showed very similar results (see supplementary Fig. S1 and Table S1).

**Fig. 3:**
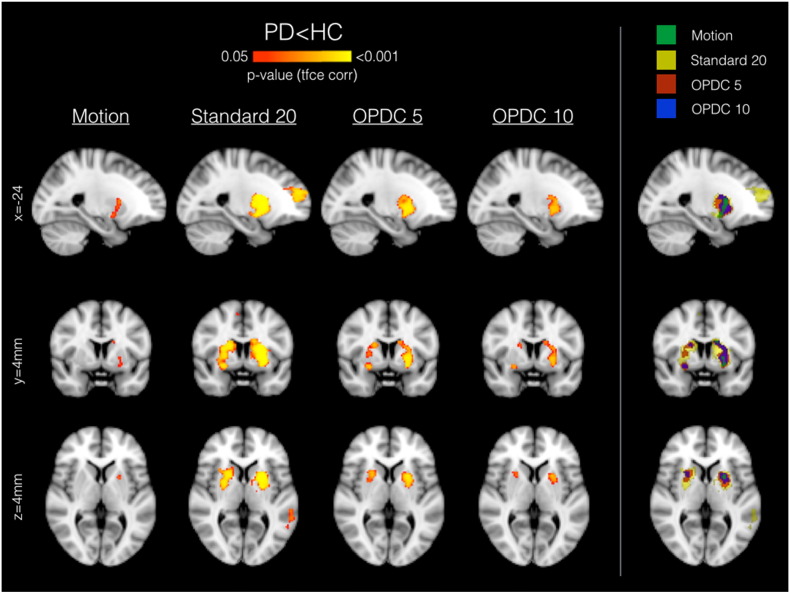
Voxel-wise between-group differences in the BGN (PD < HC) using different automated cleaning approaches on the full sample (30HC vs 59 PD). Each map is independently corrected for multiple comparisons using the TFCE approach. No significant differences were found using the opposite contrast (PD > HC).

Regarding voxel-wise results on the whole-brain BGN maps, the only significant results, corrected for multiple comparisons, were obtained with the Standard 20 option (Szewczyk-Krolikowski et al., 2014), although a similar pattern of between-group difference in FC was observed for the other ICA-based dataset at uncorrected threshold. The comparison across (automated) cleanings on the full sample confirmed this trend (see Fig. 3), showing no significant differences on uncleaned data, significant FC decrease in PD patients only in the left putamen after motion correction, and bilateral FC decrease in PD after FIX cleaning, using any of the options tested, with strongest results using the Standard 20 option.

Based on these results, the dataset cleaned with FIX, using the study-specific training dataset and a threshold of 10 (FIX OPDC 10), was chosen for subsequent analyses.

### Influence of template for dual regression

**Fig. 4:**
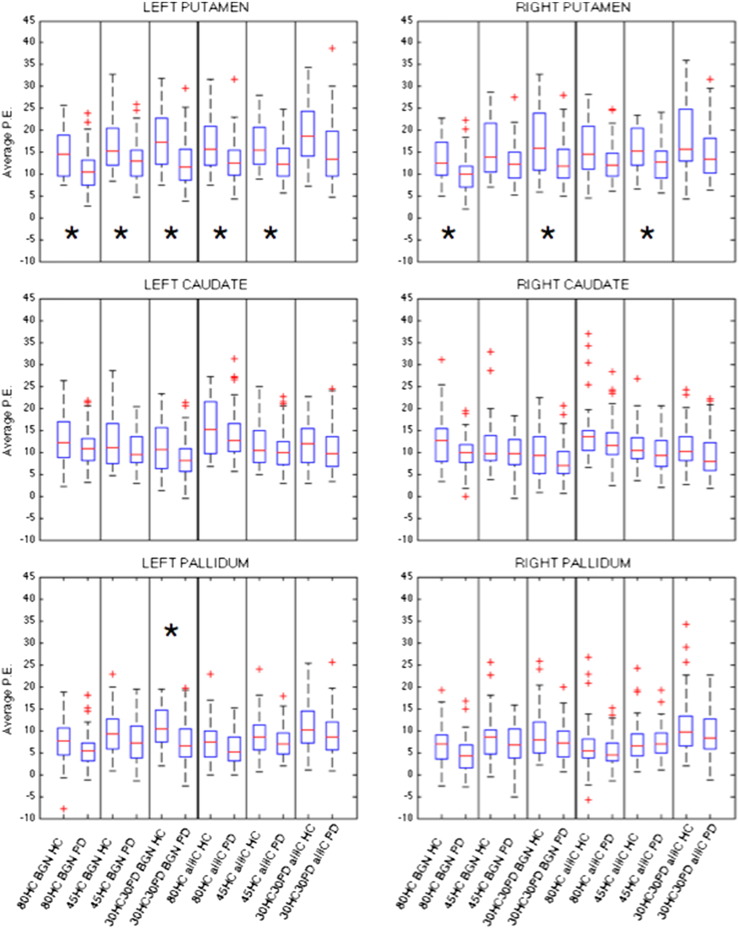
Average P.E. in the basal ganglia ROIs extracted from single-subject BGN maps obtained using different templates for dual regression. *Significant between-group differences surviving Bonferroni correction across structures.

The results of the ROI analysis in the basal ganglia (Fig. 4 and Table 2) are in line with the results obtained in the subsample, with the main between-group difference localised in the putamen. Notably, no difference was found using the 30HC30PD-ALL template.

**Fig. 5:**
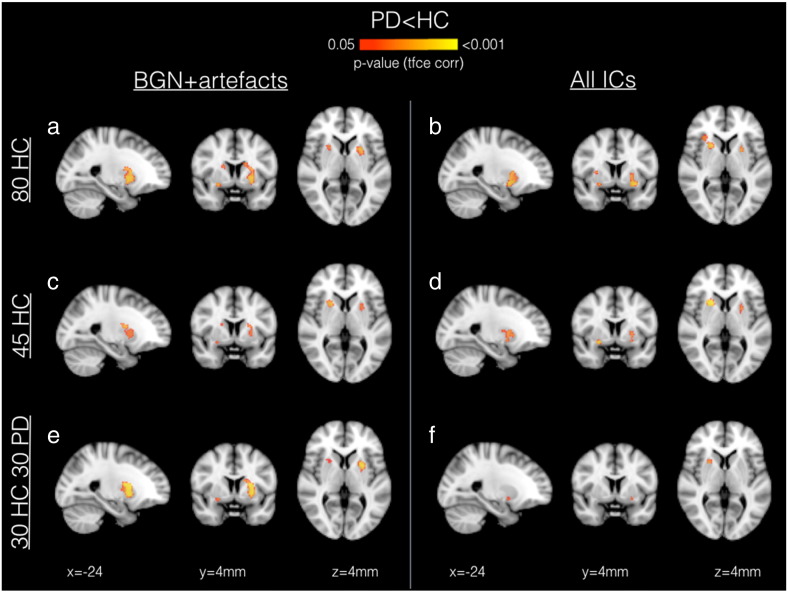
Voxel-wise between-group differences in the BGN (PD < HC) using different templates for dual regression. Each map is independently corrected for multiple comparisons using the TFCE approach. No significant differences were found using the opposite contrast (PD > HC).

Fig. 5 illustrates the results of the voxel-wise analysis: significant differences in the BGN (PD < HC, p < 0.05 TFCE corrected) were found using all templates, with the clusters mainly localised in the bilateral putamen. No significant differences were found using the opposite contrast (PD > HC).

**Fig. 6:**
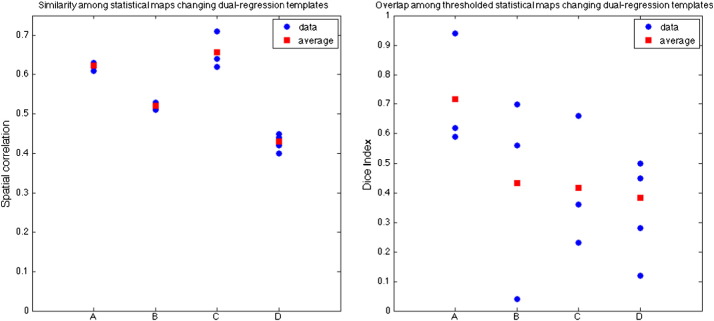
Similarity (spatial correlation of t-maps) and overlap (Dice index on thresholded t-maps) among voxel-wise analyses in the BGN using different templates for dual regression when A) changing subjects, using a subset of components including BGN and noise (Fig. 4. a–c, a–e, c–e); B) changing the set of components (subset vs all), but using the same subjects to generate the template (Fig. 4. a–b, c–d, e–f); C) changing subjects, using all components (Fig. 4. b–d, b–f, d–f); D) changing subjects and set of components (Fig. 4. other pairs).

The spatial correlation coefficients and the Dice indices reported in Fig. 6 measure the similarity among the different statistical maps (t-maps) and the overlap of the significant clusters, respectively. It can be observed that the highest similarity is obtained when changing the subjects used to generate the template (A and C), but, between these two options, the overlap of the significant clusters is much higher when using a subset of components including the BGN and the artefacts (A). Changing from using a subset of components to using all the components with the same subjects (B) decreases the spatial correlation (with respect to A and C), but on average, the overlap of the significant clusters is comparable to option C. The lowest similarity and overlap is obtained changing both the subjects and the set of components (D).

The results of the exploratory analysis on other RSNs are reported in Supplemental Table S2. When using different templates, we did not observe the same between-group differences in the same RSNs (except for the BGN); however, the results show some similarities, for example, a reduced FC in the PD patients in the right insula (see supplementary results for details).

## Discussion

The need to prove the reliability and reproducibility of scientific findings has been recently highlighted in the scientific community (Russell, 2013). This is certainly a big challenge in neuroimaging studies and, especially, in clinical studies, where the aim is to translate scientific findings in the clinical setting. Only when sufficient reproducibility is possible we will be able to produce reliable predictive, diagnostic, and prognostic biomarkers.

Typically, the analysis pipeline is one of the main contributors to result variability. Therefore, in order to allow comparison and replication, the complete disclosure of analysis parameters is crucial (Lin et al., 2012, Ridgway et al., 2008). Moreover, validation studies, such as those seen in structural MRI, are necessary whenever a new technique is introduced (Duan et al., 2015, Franco et al., 2013, Lawrenz et al., 2015, Zuo et al., 2010, Zuo and Xing, 2014), or the settings of an existing method are modified (Goto et al., 2015, Landman et al., 2007, Radua et al., 2014).

Among the different MRI modalities, functional MRI analysis techniques, and especially resting state fMRI, are the newest and most under development, as they are showing promising results in several pathologies (Barkhof et al., 2014). Due to the continuous development of new analysis methods, standard analysis pipelines or set guidelines are not currently available, preventing good reproducibility of clinical findings.

In this context, our work described the influence on the reproducibility of clinical studies of two processing steps (artefact removal and dual regression template selection) that are becoming commonly used in rfMRI analysis pipelines, but in our opinion not systematically or sufficiently analysed yet. In this way, we wanted to show the possible variability associated with these settings and to provide some guidelines to choose the right option for a clinical study and report the analysis details in scientific publications to make a study truly comparable and replicable.

To this aim, we tested the reproducibility of our recent findings regarding the functional connectivity of the basal ganglia network (BGN) in early PD patients (Szewczyk-Krolikowski et al., 2014), being particularly interested in the effect of the analysis settings in a translational neuroimaging perspective.

On the first subsample of subjects, the same used in our previous study, we tested the influence of different cleaning approaches (motion regression and different options of ICA-based cleaning) on the detection of the functional connectivity alteration in PD within the BGN. The strong between-group difference within the putamen obtained with the ROI analysis was not influenced by the cleaning approach, while small differences found in the caudate and the pallidum were enhanced or weakened depending on the approach used. Conversely, voxel-wise results were more influenced by small changes in the preprocessing than the ROI analyses. This was mainly due to the relatively low sample size, as the pattern of both uncorrected maps in the small sample and corrected maps in the full sample were similar to our previous results. The removal of more degrees of freedom when applying a more aggressive cleaning method can cause loss of statistical power, but the higher temporal SNR, higher correlation with the manually cleaned data and the results obtained on a bigger sample suggest that the use of ICA-based cleaning can help to increase the reproducibility of clinical findings, as it removes non neural-related sources of inter-individual difference. FIX was always run using the “soft” option, i.e. removing only the unique variance related to the artefacts (see Griffanti et al., 2014, for details). The “aggressive” option available with the tool was also tested, but given that we obtained very similar results, we did not included them in this paper, to limit the total number of approaches compared.

Interestingly, the strongest results were obtained with FIX trained with the standard training dataset instead of the study-specific one (OPDC). From Fig. 2 and Table 1, it can be observed that cleaning reduces within-group variance in both patients and controls; however, the amount of variance removed (decrease in standard deviation) is higher in HC than PD. This suggests that cleaning is removing more non-neuronal fluctuation in the HC group. The fact that the amount of head motion was higher (although not significantly) in the HC than in the PD (which are predominantly patients with akinetic-rigid parkinsonism with minimal tremor) raises the possibility that the group-dependent non-neuronal fluctuation is due to motion. However, the removal of motion parameters did not improve the detection of the BGN alteration in PD as much as the ICA-based options, which remove multiple sources of noise. Re-running the voxel-wise analyses on the full sample including the average relative head motion as an additional covariate produced very similar results (results not shown). Therefore, we suggest that i) we have provided additional evidence in line with current literature (Murphy et al., 2013) that motion regression is important, but not sufficient to effectively remove noise when performing rfMRI analyses; ii) the difference observed between PD and HC is not driven by differences in head motion (although we cannot exclude that residual motion could contribute to the FC differences).

We also observed that both before and after cleaning, the variability of the FC in the BGN is lower in the PD than the HC. We speculate that this could be due to the fact that a diseased population is more homogeneous than a healthy population, especially in a brain region known to be affected by the pathology. In the healthy subjects instead, many more factors can influence the FC, both biological and artefact related. Of the options tested, we selected an ICA-based cleaning (FIX) trained on study-specific data and with a threshold of 10, which showed good results both in terms of training dataset accuracy (TPR/TNR) and similarity with the results obtained using manual cleaning, our gold standard method. We speculate that the difference between this method and the one that gave the most significant results (Standard 20) could be due to the fact that the study-specific training dataset better reduced the noise-related variability, especially in the HC, while preserving the biologically meaningful variance, which remains higher in HC than in the PD group after cleaning, but allows to detect the pathological alteration in the PD group.

Our second aim was to test the influence of the use of 6 different templates for dual regression, generated by changing the number and type of subjects included and/or the number of components used in the set of spatial regressors. The results of the ROI analysis are fairly consistent across templates and in line with the results obtained in the smaller sample, so also stable when increasing the sample size. From the voxel-wise analysis of the BGN, we obtained similar results across templates showing a reduced FC in the PD patients in the bilateral putamen, in concordance the ROI analysis. Interestingly, the weakest results were obtained with the 30HC30PD template. Although the use of a balanced number of patients and controls to run group-ICA and obtain a study-specific template is a common practice, as it avoids the template being biassed toward one group, this might not be the most suitable option for a clinical study including patients and controls. In fact, the aim of such a study is to detect differences relative to a healthy brain, rather than differences between any of the two groups and an “average brain”, without being influenced by the characteristics of the experimental population (Schultz et al., 2014). Therefore, the use of an out-of-sample set of HC (i.e. not including the ones used to test the between-group differences) to build the template used for dual regression is recommended for such clinical studies.

The differences among the other templates can be explained by looking at the results of the similarity and overlap across the thresholded statistical maps. When using only the BGN (plus artefactual components) in the dual regression, the results were much less influenced by the number/type of subjects used to generate the template. This is because the BGN map is similar across templates (spatial correlation coefficient between the BGN component of the template: 80HC–45HC = 0.83; 80HC–30HC30PD = 0.78; 45HC–30HC30PD = 0.76), and the artefactual components include similar sources of structured noise (WM, CSF, blood vessels, residual motion). Either changing only the subjects when using all the components or changing only the approach when using the same subjects seems to have the same influence on the overlap among significant clusters (Fig. 5 B vs C, Dice index). However, the spatial correlation among the t-maps is higher when changing the subjects but not the approach. In both cases, new or different components are introduced and the variance of the data is distributed in a different way across the regressors, but changing the subset of components introduces more variability in the results than changing the subjects included in the template while generating the template with the same approach. As expected, changing both options induces the highest variability in the results.

We chose to test the reproducibility of the difference in BG connectivity between PD and HC because, in our previous study, we observed it to be substantial in both magnitude and extent and thus provided a good test-bed. The results become much more variable when considering a higher number of components i.e. when performing more exploratory analyses. As described in the supplementary material, when we tested between-group differences in other RSNs, there was not complete agreement on the results obtained with the three templates (80HC-ALL, 45HC-ALL, and 30HC30PD-ALL), except for the BGN, although a few similarities were observed. For example, an alteration of the insula, which has been suggested to play a role in the non-motor symptoms of Parkinson's disease (Christopher et al., 2014), was identified with two templates, although in different components. This suggests that exploratory analyses (e.g. testing multiple RSNs) should be used to formulate new hypotheses, but careful validation is necessary before biomarkers can be widely applied. In our case, the differences found in the insula can be subject of future studies to explore in more detail the involvement of this brain region in the pathology.

## Conclusion

To conclude, our study systematically delineates the influence of artefact removal and the choice of the template for dual regression on the reproducibility of clinical findings with rfMRI, providing some guidelines to obtain more reliable results. Being aware of the differences introduced by analysis choices can help to compare different studies and to decide the most suitable approach for a particular research question, being conscious of the possible variability/bias introduced or avoided. A detailed description of the analysis details in scientific publications is also needed so that studies can be compared and reproduced, toward the definition of reliable imaging clinical biomarkers.

## Figures and Tables

**Table 1:** ROI analysis for different cleaning approaches. Group comparison of average P.E. in the basal ganglia ROIs extracted from single-subject BGN maps.

	Average P.E. in the BGN		Group comparison HC vs PD		Average P.E. in the BGN		Group comparison HC vs PD	
	HC (mean ± stdev)	PD (mean ± stdev)	t-value	p-value⁎	HC (mean ± stdev)	PD (mean ± stdev)	t-value	p-value⁎
	Left putamen				Right putamen			
Uncleaned	23.38 ± 9.06	16.05 ± 7.36	2.739	0.01	22.61 ± 8.65	17.08 ± 7.02	2.162	0.037
Motion	19.06 ± 7.79	11.61 ± 5.29	3.444	0.001⁎	17.92 ± 6.94	12.61 ± 5.06	2.695	0.011
Standard 20	16.65 ± 6.20	10.00 ± 4.03	3.92	< 0.001⁎	16.01 ± 6.25	10.14 ± 3.82	3.493	0.002⁎
OPDC 5	15.27 ± 5.57	10.15 ± 3.33	3.441	0.002⁎	15.18 ± 6.29	10.15 ± 3.42	3.062	0.005⁎
OPDC 10	14.53 ± 5.44	9.62 ± 3.28	3.366	0.002⁎	14.19 ± 5.53	9.65 ± 3.40	3.046	0.005⁎
Manual	11.73 ± 5.10	7.54 ± 2.88	3.116	0.004⁎	11.36 ± 4.71	7.69 ± 2.26	3.06	0.005⁎

	Left caudate				Right caudate			
Uncleaned	20.82 ± 7.85	17.81 ± 6.92	1.252	0.219	19.68 ± 10.07	15.88 ± 8.51	1.258	0.216
Motion	16.73 ± 7.22	11.96 ± 5.18	2.341	0.025	15.73 ± 8.48	10.31 ± 5.98	2.277	0.029
Standard 20	14.70 ± 6.86	9.56 ± 4.22	2.782	0.009	14.43 ± 7.61	7.60 ± 4.46	3.371	0.002⁎
OPDC 5	13.90 ± 6.68	10.25 ± 3.91	2.052	0.049	13.35 ± 6.96	8.17 ± 4.33	2.755	0.009
OPDC 10	13.27 ± 6.65	9.74 ± 3.54	2.041	0.051	12.58 ± 7.18	7.85 ± 3.98	2.513	0.017
Manual	10.87 ± 5.37	7.82 ± 2.57	2.229	0.035	10.30 ± 5.47	6.46 ± 3.30	2.625	0.013

	Left pallidum				Right pallidum			
Uncleaned	13.10 ± 7.27	7.48 ± 5.73	2.646	0.012	12.75 ± 8.76	8.47 ± 4.64	1.882	0.068
Motion	10.49 ± 5.54	5.42 ± 4.85	3.001	0.005⁎	10.51 ± 6.81	6.05 ± 3.78	2.494	0.017
Standard 20	8.68 ± 4.55	4.79 ± 2.76	3.192	0.003⁎	8.67 ± 6.41	4.66 ± 2.96	2.478	0.02
OPDC 5	7.37 ± 5.04	5.01 ± 2.73	1.798	0.081	7.86 ± 6.29	4.67 ± 2.88	2.008	0.052
OPDC 10	7.18 ± 5.56	4.63 ± 2.81	1.783	0.083	7.28 ± 4.93	4.20 ± 2.85	2.358	0.024
Manual	5.35 ± 4.70	3.74 ± 2.60	1.301	0.201	5.07 ± 3.46	3.34 ± 2.21	1.831	0.075

⁎ Significant after correction for multiple comparisons across 6 structures.

**Table 2:** ROI analysis for different templates for dual regression. Group comparison of average P.E. in the basal ganglia ROIs extracted from single-subject BGN maps.

	Average P.E. in the BGN		Group comparison HC vs PD		Average P.E. in the BGN		Group comparison HC vs PD	
	HC (mean ± stdev)	PD (mean ± stdev)	t-value	p-value⁎	HC (mean ± stdev)	PD (mean ± stdev)	t-value	p-value⁎
Left putamen					Right putamen			
80 HC BGN	14.62 ± 5.38	10.79 ± 4.35	3.374	0.001⁎	13.38 ± 5.29	10.09 ± 4.24	3.18	0.002⁎
45 HC BGN	16.64 ± 5.99	12.76 ± 4.50	3.131	0.003⁎	15.66 ± 6.32	12.65 ± 4.39	2.335	0.024
30HC30PD BGN	17.77 ± 6.53	12.85 ± 5.65	3.681	< 0.001⁎	17.45 ± 7.76	12.73 ± 5.50	2.973	0.005⁎
80 HC allIC	17.15 ± 6.93	13.07 ± 4.89	2.876	0.006⁎	15.66 ± 6.00	12.32 ± 4.13	2.74	0.009
45 HC allIC	16.64 ± 5.40	12.89 ± 4.28	3.565	0.001⁎	15.72 ± 4.96	12.67 ± 3.96	3.144	0.002⁎
30HC30PD allIC	19.11 ± 7.69	14.93 ± 6.91	2.597	0.011	18.29 ± 7.46	14.44 ± 5.95	2.649	0.01

Left caudate					Right caudate			
80 HC BGN	13.36 ± 6.03	10.77 ± 3.88	2.138	0.038	12.89 ± 6.14	9.92 ± 3.75	2.429	0.02
45 HC BGN	12.36 ± 5.94	10.61 ± 4.30	1.433	0.159	11.81 ± 6.17	9.94 ± 3.99	1.726	0.088
30HC30PD BGN	11.00 ± 6.29	8.34 ± 4.41	2.071	0.044	9.47 ± 5.59	7.51 ± 4.27	1.831	0.071
80 HC allIC	15.90 ± 6.66	13.89 ± 5.62	1.496	0.138	14.87 ± 7.59	12.67 ± 5.05	1.628	0.107
45 HC allIC	11.57 ± 5.04	10.39 ± 4.59	1.106	0.272	11.40 ± 4.71	9.72 ± 4.27	1.692	0.094
30HC30PD allIC	11.91 ± 5.42	10.83 ± 5.12	0.925	0.357	11.37 ± 5.42	9.61 ± 4.94	1.532	0.129

Left pallidum					Right pallidum			
80 HC BGN	7.57 ± 5.49	5.44 ± 3.73	1.912	0.062	7.05 ± 4.65	4.65 ± 4.15	2.476	0.015
45 HC BGN	9.85 ± 5.12	7.61 ± 4.43	2.144	0.035	8.87 ± 5.89	7.11 ± 4.51	1.563	0.122
30HC30PD BGN	10.61 ± 4.63	7.26 ± 4.74	3.179	0.002⁎	9.62 ± 6.25	7.24 ± 4.30	2.105	0.038
80 HC allIC	7.78 ± 5.26	5.81 ± 3.46	1.862	0.07	6.62 ± 6.88	5.25 ± 3.74	1.015	0.316
45 HC allIC	9.10 ± 4.93	7.49 ± 3.45	1.792	0.077	7.66 ± 5.54	7.23 ± 3.75	0.433	0.666
30HC30PD allIC	11.27 ± 6.06	9.00 ± 4.97	1.892	0.062	11.47 ± 7.74	9.11 ± 4.92	1.747	0.084

⁎ Significant after correction for multiple comparisons across 6 structures.
