# Supplementary material for: Challenges in the reproducibility of clinical studies with resting state fMRI: An example in early Parkinson's disease
Source: Neuroimage. 2016 Jan 1;124(Pt A):704–13. doi: 10.1016/j.neuroimage.2015.09.021 (PMC4655939; doi:10.1016/j.neuroimage.2015.09.021)
Supplement: Supplementary file 1 — Supplementary materials [file mmc1.docx]

**SUPPLEMENTARY MATERIAL**

**Challenges in the reproducibility of clinical studies with resting state fMRI: an example in early Parkinson’s disease**

Ludovica Griffanti^1^, Michal Rolinski^2,3^, Konrad Szewczyk-Krolikowski^2,3^, Ricarda A. Menke^1^, Nicola Filippini^1,4^, Giovanna Zamboni^1^, Mark Jenkinson^1^, Michele T.M. Hu^2,3^, Clare E. Mackay^1,2,4*^

1. Centre for the functional MRI of the Brain (FMRIB), University of Oxford
2. Oxford Parkinson’s Disease Centre (OPDC)
3. Nuffield Department of Clinical Neurosciences, University of Oxford
4. Department of Psychiatry, University of Oxford

**Methods**

*Influence of template for dual regression on the reproducibility of FC within other RSNs*

In order to investigate if and how changing the template used for dual regression would affect also the results in other RSNs besides the BGN, we performed an exploratory analysis. On the components identified as not artefactual in templates 80HC-ALL, 45HC-ALL, and 30HC30PD-ALL, we performed voxel-wise non-parametric permutation test (p=0.05 corrected for multiple comparisons with TFCE approach, minimum cluster size 35 voxels), covarying for age and voxel-wise GM, and compared the results across templates.

*Reproducibility regarding groups’ composition*

In order to test the impact of the (automated) cleaning methods and the templates on the reproducibility of results using different datasets, we randomly split the full sample into two group pairs of PD patients and HC and repeated the analyses over 100 permutations regarding groups’ composition.

As the sample size when splitting the sample in half was quite low (15 HC vs 29 or 30 PD), the voxel-wise analyses did not have enough power to show significant between group differences (as in the subsample used to initially compare 19 HC vs 19 PD). We therefore focused on the parameters estimate (P.E.) values extracted from the ROI analysis. On those data we:

1. randomly split the sample in two group pairs (A and B) of patients vs controls 100 times.
2. performed a 2 sample independent t-test between patients and controls for group A and group B separately and obtained a t-value for each test (t-value(A) and t-value(B)) for each of the 100 realizations.
3. calculated the absolute difference between the two t-values (Delta t-value = abs (t-value(A) – t-value(B)). This was done because we are interested in the results to be as similar as possible (regardless the significance or the directionality of the difference).
4. plotted Delta t-value across the 100 realizations for each structure, comparing (i) the different (automated) cleaning option, and (ii) the different templates. Higher Delta t-value corresponds to lower reproducibility across datasets.

**Results**

*Influence of template for dual regression on the reproducibility of FC within other RSNs*

From the analyses of all the RSNs we identified 29 components as physiologically meaningful (i.e. neither artefactual nor belonging to the cerebellum, due to the small field of view) in the 80HC-ALL template, 27 the 45HC-ALL template and 25 in the 30HC30PD-ALL template. Among those components, we found significant differences in 5 RSNs using 80HC-ALL, in 4 RSNs with 45HC-ALL, and in 4 RSNs with 30HC30PD-ALL (see supplemental Table S2 for details). The results did not show the same between-group differences in the same RSNs (except for the BGN), however the results show some similarities. For example, a reduced FC in the PD patients in the right insula was observed in the BGN component with the 80HC-ALL template and in the salience network component using the 45HC-ALL template. Moreover, except for the alteration in the right putamen observed with the 45HC-ALL template, none of the other clusters survived correction for multiple comparisons across components (i.e. 29 when using 80HC-ALL, 27 for 45HC-ALL and 25 for 30HC30PD-ALL).

*Reproducibility regarding groups’ composition*

Figure S2 shows the results for the different cleaning options: the difference in t-values is very similar across options. In the putamen (which showed the most significant results in the full sample), the Standard 20 option is marginally less reproducible. This approach was the one which showed the strongest voxel-wise results, but less correlation with the manual cleaning and less variance removed by FIX (compared to OPDC 5 and OPDC 10). We therefore speculate that the variance in the BOLD signal that was not removed by FIX causes a loss of reproducibility.

Figure S3 shows the same results for the different templates. In this case the results are even more similar across options, showing that all the approaches we tested are quite stable with respect to reproducibility across datasets.

**SUPPLEMENTARY FIGURES**

**
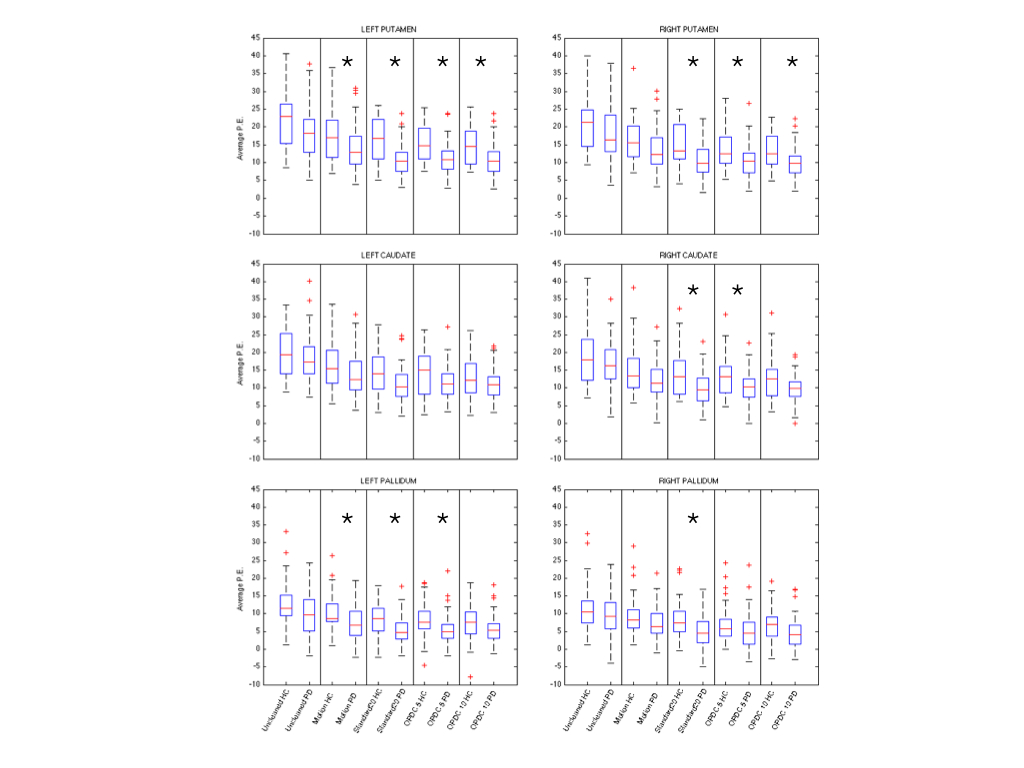
**

Figure S1. Average P.E. in the basal ganglia ROIs extracted from single subject BGN maps obtained from data of the full sample (30 HC vs 59 PD) cleaned with different automated options. *Significant between-group differences surviving Bonferroni correction across structures.


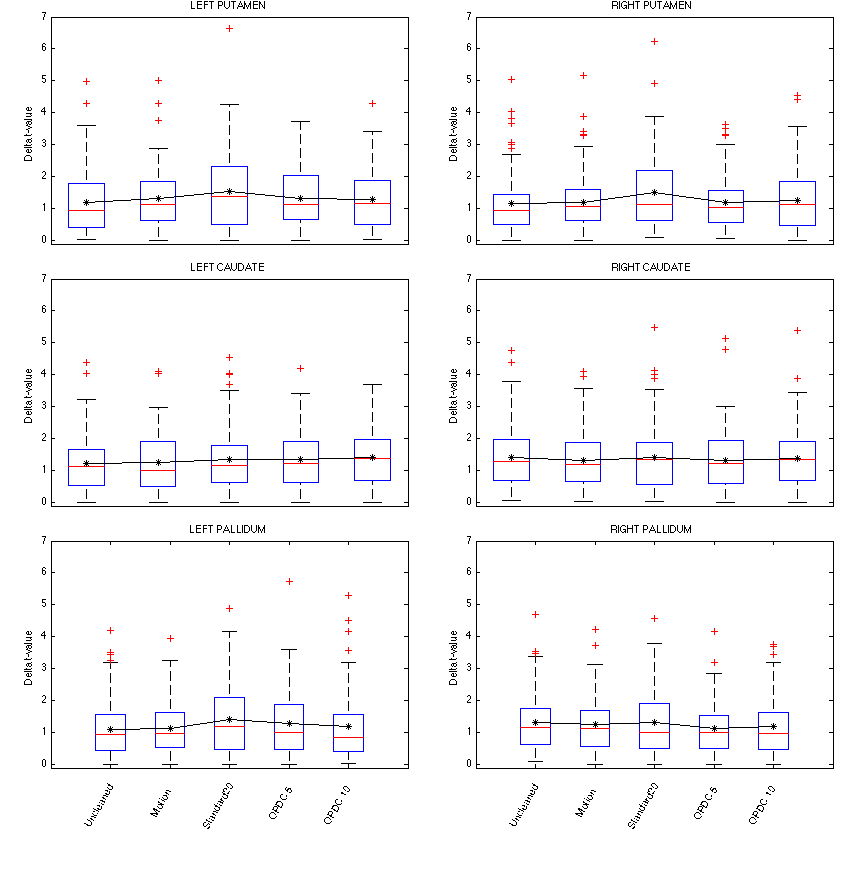


Figure S2. Boxplot of Delta t-value across 100 realizations for each structure and (automated) cleaning method. The average values are also plotted in black. Delta t-value = abs (t-value(A) – t-value(B)), where t-value(A) and t-value(B) are the results of the 2 sample independent t-test between patients and controls after randomly splitting the sample in two groups of patients and controls A and B.


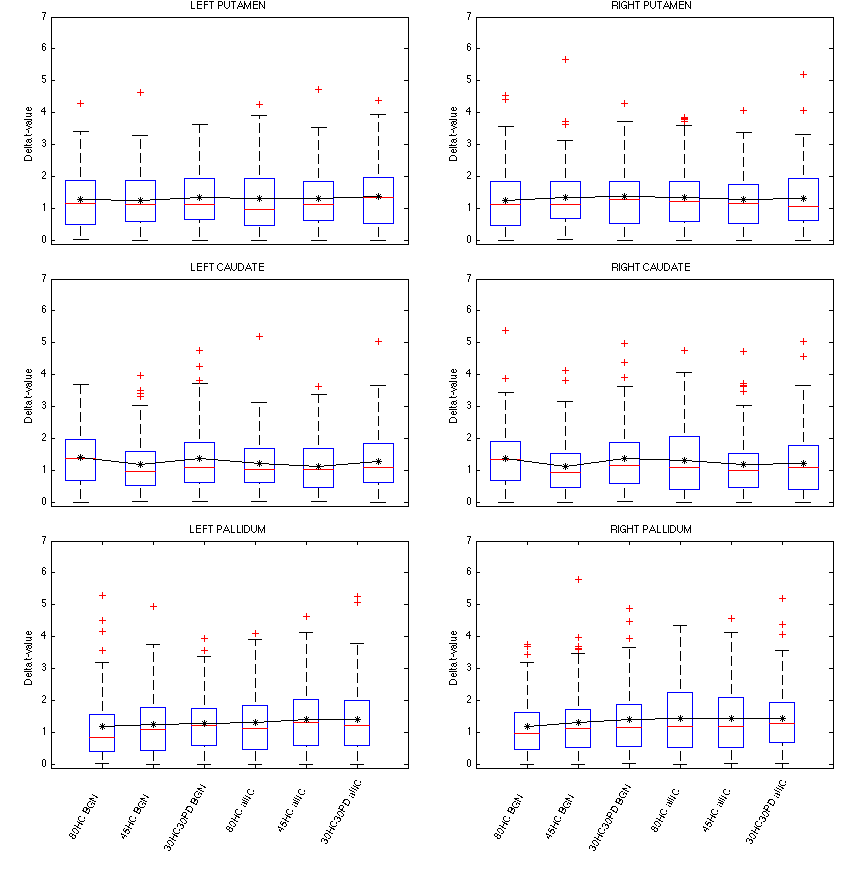


Figure S3. Boxplot of Delta t-value across 100 realizations for each structure and template for dual regression. The average values are also plotted in black. Delta t-value = abs (t-value(A) – t-value(B)), where t-value(A) and t-value(B) are the results of the 2 sample independent t-test between patients and controls after randomly splitting the sample in two groups of patients and controls A and B.

**SUPPLEMENTARY TABLES**

**Table S1.** ROI analysis for different cleaning approaches. Group comparison of average P.E. in the basal ganglia ROIs extracted from single subject BGN maps obtained from data of the full sample (30 HC vs 59 PD).

|  | Average P.E. in the BGN | | Group comparisonHC vs PD | | Average P.E. in the BGN | | Group comparisonHC vs PD | |
| --- | --- | --- | --- | --- | --- | --- | --- | --- |
|  | HC (mean±stdev) | PD (mean±stdev) | t-value | p-value | HC (mean±stdev) | PD (mean±stdev) | t-value | p-value |
|  | LEFT PUTAMEN | |  |  | RIGHT PUTAMEN | |  |  |
| Uncleaned | 21.92±8.05 | 18.07±7.56 | 2.225 | 0.029 | 21.11±7.57 | 18.07±7.21 | 1.85 | 0.068 |
| Motion | 17.91±6.95 | 13.82±6.11 | 2.846 | 0.006* | 16.77±6.25 | 13.90±5.75 | 2.159 | 0.034 |
| Standard 20 | 16.15±6.02 | 10.91±4.43 | 4.219 | <0.001* | 14.88±6.03 | 10.56±4.27 | 3.506 | 0.001* |
| OPDC 5 | 15.41±5.70 | 10.95±4.32 | 3.776 | <0.001* | 14.31±6.23 | 10.42±4.57 | 3.029 | 0.004* |
| OPDC 10 | 14.62±5.38 | 10.79±4.35 | 3.374 | 0.001* | 13.38±5.29 | 10.09±4.24 | 3.18 | 0.002* |
|  | LEFT CAUDATE | |  |  | RIGHT CAUDATE | |  |  |
| Uncleaned | 20.36±7.22 | 18.39±6.66 | 1.279 | 0.204 | 18.99±8.58 | 16.57±6.22 | 1.519 | 0.132 |
| Motion | 16.15±6.54 | 13.67±5.99 | 1.791 | 0.077 | 15.14±7.17 | 12.29±5.23 | 2.138 | 0.035 |
| Standard 20 | 14.45±6.30 | 10.92±4.64 | 2.717 | 0.009 | 14.13±6.56 | 9.63±4.30 | 3.402 | 0.001* |
| OPDC 5 | 14.14±6.26 | 11.45±4.20 | 2.119 | 0.04 | 13.73±6.09 | 10.16±4.25 | 2.876 | 0.006* |
| OPDC 10 | 13.36±6.03 | 10.77±3.88 | 2.138 | 0.038 | 12.89±6.14 | 9.92±3.75 | 2.429 | 0.02 |
|  | LEFT PALLIDUM | |  |  | RIGHT PALLIDUM | |  |  |
| Uncleaned | 12.76±6.65 | 9.75±6.19 | 2.114 | 0.037 | 11.58±7.01 | 9.76±5.97 | 1.285 | 0.202 |
| Motion | 10.21±5.38 | 7.08±4.99 | 2.724 | 0.008 | 9.66±6.16 | 7.49±4.76 | 1.841 | 0.069 |
| Standard 20 | 8.98±4.54 | 5.37±3.73 | 4.007 | <0.001* | 8.63±5.92 | 5.24±4.25 | 3.107 | 0.003* |
| OPDC 5 | 8.13±5.25 | 5.36±3.93 | 2.804 | 0.006* | 7.54±5.79 | 4.96±4.64 | 2.276 | 0.025 |
| OPDC 10 | 7.57±5.49 | 5.44±3.73 | 1.912 | 0.062 | 7.05±4.65 | 4.65±4.15 | 2.476 | 0.015 |

*Significant after correction for multiple comparisons across 6 structures

**Table S2.** Areas showing significant between-group difference in FC in the RSNs identified for each template.

| **Template** | **Resting State Network** | **contrast** | **max corrp** | **Areas of significant difference** |
| --- | --- | --- | --- | --- |
| 80HC-ALL | Basal Ganglia Network (BGN) | PD<HC | 0.011 | Bilateral Putamen, Right Insula |
|  | Sensory-Motor Network (SMN) | PD<HC | 0.004 | SMN |
|  | Medial Temporal | PD<HC | 0.007 | Left Hippocampus |
|  | Task Positive | PD>HC | 0.001 | Right Superior Parietal gyrus |
|  | Left SMN | PD<HC | 0.003 | Left Parietal lobe |
| 45HC-ALL | BGN | PD<HC | 0.002* | Bilateral Putamen |
|  | Superior frontal | PD<HC | 0.017 | Inferior Frontal gyrus |
|  | Salience Network (mainly Insula) | PD<HC | 0.006 | Right Insula |
|  | Ventral Stream (temporal poles) | PD<HC | 0.011 | Right Temporal pole |
| 30HC30PD-ALL | BGN | PD<HC | 0.021 | Bilateral Putamen |
|  | Lateral Left | PD<HC | 0.020 | Left Superior Parietal lobe |
|  | Ventral Stream (temporal pole) | PD<HC | 0.010 | Right Hippocampus, Right Temporal Pole, Right Putamen |
|  | SMN | PD<HC | 0.007 | Left Lateral Occipital lobe |

*survives correction for multiple components.
